# Illustration of Gut–Thyroid Axis in Alcohol Use Disorder: Interplay of Gut Dysfunction, Pro-Inflammatory Responses, and Thyroid Function

**DOI:** 10.3390/cells11193100

**Published:** 2022-10-01

**Authors:** Manasa Sagaram, Amor J. Royer, Huirong Hu, Abhas Rajhans, Ranganathan Parthasarathy, Sathya Sridevi Krishnasamy, Sri Prakash Mokshagundam, Maiying Kong, Melanie L. Schwandt, Dipendra Parajuli, Matthew C. Cave, Vatsalya Vatsalya

**Affiliations:** 1 Division of Gastroenterology, Hepatology and Nutrition, Department of Medicine, University of Louisville, Louisville, KY 40202, USA; 2 Clinical Laboratory for the Intervention Development of AUD and Organ Severity, University of Louisville, Louisville, KY 40202, USA; 3 Department of Bioinformatics and Biostatistics, University of Louisville, Louisville, KY 40202, USA; 4 Department of Neuroscience, University of California Los Angeles, Los Angeles, CA 90095, USA; 5 Division of Endocrinology, Metabolism & Diabetes, University of Louisville, Louisville, KY 40202, USA; 6 University of Louisville Alcohol Research Center, University of Louisville, Louisville, KY 40202, USA; 7 National Institute on Alcohol Abuse and Alcoholism, Bethesda, MD 20892, USA; 8 Robley Rex VA Medical Center, Louisville, KY 40206, USA

**Keywords:** alcohol use disorder, gut dysfunction, gut–thyroid axis, pro-inflammatory cytokines, thyroid-associated hormones

## Abstract

(1) Background: Heavy and chronic alcohol drinking leads to altered gut dysfunction, coupled with a pro-inflammatory state. Thyroid-associated hormones and proteins may be dysregulated by heavy and chronic alcohol intake; however, the mechanism for altered gut-derived changes in thyroid function has not been studied thus far. This study investigates the role of alcohol-induced gut dysfunction and pro-inflammatory cytokine profile in the thyroid function of patients with alcohol use disorder (AUD). (2) Methods: Male and female AUD patients (*n* = 44) were divided into Gr.1, patients with normal thyroid-stimulating hormone (TSH) levels (*n* = 28, 0.8 ≤ TSH ≤ 3 mIU/L); and Gr.2, patients with clinically elevated TSH levels (*n* = 16, TSH > 3 mIU/L). Demographics, drinking measures, comprehensive metabolic panels, and candidate thyroid markers (TSH, circulating triiodothyronine (T3), and free thyroxine (fT4)) were analyzed. Gut-dysfunction-associated markers (lipopolysaccharide (LPS), LPS-binding protein (LBP), and soluble LPS-induced pathogen-associated protein (sCD14)), and candidate pro-inflammatory cytokines (IL-1β, TNF-α, IL-6, IL-8, MCP-1, PAI-1) were also evaluated. (3) Results: Patients in both groups presented with a borderline overweight BMI category. Gr.2 reported numerically higher indices of chronic and heavy drinking patterns than Gr.1. The fT4 levels were elevated, while T3 was within normal limits in both groups. The gut dysfunction markers LBP and sCD14 were numerically elevated in Gr.2 vs. Gr.1, suggesting subtle ongoing changes. Candidate pro-inflammatory cytokines were significantly elevated in Gr.2, including IL-1 β, MCP-1, and PAI-1. Gr.2 showed a strong and statistically significant effect on the gut–immune–thyroid response (r = 0.896, 36 *p* = 0.002) on TSH levels in a multivariate regression model with LBP, sCD14, and PAI-1 levels as upstream variables in the gut–thyroid pathway. In addition, AUROC analysis demonstrated that many of the cytokines strongly predicted TSH in Gr.2, including IL-6 (area = 0.774, 39 *p* < 0.001) and TNF-α (area = 0.708, *p* = 0.017), among others. This was not observed in Gr.1. Gr.2 demonstrated elevated fT4, as well as TSH, which suggests that there was subclinical thyroiditis with underlying CNS dysfunction and a lack of a negative feedback loop. (4) Conclusions: These findings reveal the toxic effects of heavy and chronic drinking that play a pathological role in thyroid gland dysregulation by employing the gut–brain axis. These results also emphasize potential directions to carefully evaluate thyroid dysregulation in the overall medical management of AUD.

## 1. Introduction

Alcohol use disorder (AUD) is characterized by compulsive alcohol consumption over an extended period of time, combined with a loss of control and negatively associated emotions [1,2]. According to the 2019 National Survey on Drug Use and Health, 14.1 million adults in the United States meet the criteria for an AUD diagnosis [3]. Thyroid dysfunction and its association with chronic alcohol use have been described, though its precise pathophysiology has not yet been fully ascertained. One important consideration is that there is a significant contribution from alcohol-induced gut dysfunction and concomitant pro-inflammatory cytokine release to elicit deleterious effects.

Several studies describe the effects of heavy alcohol consumption on thyroid homeostasis. This was initially described in 1960 when Goldberg suggested that a high blood alcohol content exerts direct toxic effects on the vascular thyroid. He also provided an alternative explanation for the possible toxic effects of alcohol on the thyrotropin areas of the hypothalamus or pituitary rather than the thyroid gland itself [4]. The effect of alcohol abuse on the hypothalamic–pituitary–thyroid (HPT) axis was further examined by Zoeller et al., who demonstrated that rats with chronic ethanol treatment developed significantly elevated thyrotropin-releasing hormone (TRH) and TSH levels and a blunted thyrotropic response to cold exposure [5]. Further studies have demonstrated similar findings in patients with chronic alcohol use, as well as dysregulation of the HPT axis caused by elevation of TRH or TSH with a blunted response [6,7,8,9].

The pathogenesis of this response is unknown but has been hypothesized to be either a direct effect of alcohol on the HPT axis or, more interestingly, an indirect mechanism via gut dysfunction and pro-inflammatory responses. Chronic and excessive consumption of alcohol causes gut dysfunction through microbiome dysbiosis and through an increase in intestinal permeability [10,11]. Alcohol can increase intestinal bacteria through poor digestive and intestinal function, which are secondary to alcohol consumption [10]. Alcohol use also causes dysbiosis, with a change in the ratio between beneficial and pathogenic bacteria [10,12]; these increases and alterations in the microbiome result in an increase in endotoxin production, including lipopolysaccharide (LPS), lipopolysaccharide-binding protein (LBP), and LPS-induced pathogen-associated protein (sCD14). Additionally, alcohol increases intestinal permeability by alcohol-induced direct cellular damage to gut epithelial cells, as well as disruption of tight junctions, cytoskeletons, and other proteins, leading to increased leakiness of the gut [13,14,15].

This increase in gut dysfunction, as well as alcohol metabolism in the liver, further contributes to a pro-inflammatory state in chronic alcohol use. Endotoxins, such as LPS, bind to toll-like receptor 4 (TLR-4), initiating a cascade of events; this leads to the production of reactive oxygen species (ROS), along with pro-inflammatory cytokines, including tumor necrosis factor α (TNF-α), interleukin 6 (IL-6), interleukin 8 (IL-8), interleukin 1β (IL-1β), plasminogen activator inhibitor 1 (PAI-1), and monocyte chemoattractant protein 1 (MCP-1), among others [16,17,18]. These contribute to systemic inflammation and organ dysfunction.

The complexity of alcohol-associated dysregulation in the HPT axis, as well as its relationship to gut-derived alterations, is still being unraveled in the scientific community. The aims of this study are to identify the role of gut dysfunction and a pro-inflammatory state in thyroid dysregulation in patients with AUD; to estimate the levels of dysregulation in thyroid function; and, lastly, to characterize the gut–thyroid axis for the pathological role of chronic and heavy alcohol drinking. 

## 2. Materials and Methods

### 2.1. Patient Recruitment

This clinical, cross-sectional, one timepoint study was a secondary project of a larger clinical protocol (NCT#00106106) conducted by the National Institute on Alcohol Abuse and Alcoholism (NIAAA) of the National Institutes of Health (NIH), Bethesda, MD. The overarching protocol had a primary aim of determining if acamprosate reduced alcohol craving and withdrawal symptoms in patients receiving standard inpatient care for alcohol detoxification. There were several secondary aims of this project at various time points, which included evaluating alcohol-induced gut dysfunction and its effect on the thyroid. This study protocol was Institutional Review Board (IRB) approved through the Central Neuroscience Committee of the NIAAA. The participants were screened, and eligible patients consented to participate in this study prior to the collection of bodily samples and clinical and research data.

### 2.2. Eligibility Criteria and Randomization

All study patients were diagnosed with AUD based on DSM-IV, TR edition. Altogether 44 male and female AUD patients between the ages of 23–63 years participated in this investigation. Primary exclusion criteria included: clinical diagnoses of liver disease, which included alcohol-associated hepatitis, cirrhosis, viral hepatitis, and autoimmune hepatitis; clinical diagnosis of pancreatic disease, which included acute pancreatitis, chronic pancreatitis, and pancreatic cancer; and diagnosis of a severe physical or psychiatric disease, such as unstable cardiovascular disease (with decompensation demonstrated through chest X-ray or pathological echocardiogram), renal failure with creatinine clearance <30 mL/min, and/or advanced lung disease. Other exclusion criteria included the following: a diagnosis of HIV, pregnancy, ongoing breastfeeding, use of biotin supplements, and/or positive urine drug screen for illicit substances. Further inclusion and exclusion criteria are detailed in a previous publication [19]. Thyroid-stimulating hormone (TSH) levels were used as reference factors to stratify patients into two groups—normal TSH levels between and including 0.8 and 3 mIU/L (Group 1 [Gr.1]); and TSH levels greater than 3 mIU/L (Hypothyroid, Group 2 [Gr.2]) [20,21]. It should be noted that the upper limit for normal TSH of 3 mIU/L is lower than the more commonly used 5 mIU/L. This was deliberately chosen due to several proposals that the upper limit for normal should be lowered [20,21]. One argument discusses that there is a higher level of antithyroid antibodies detected in patients who have a serum TSH between 3 and 5 mIU/L [20]. Another proposal argues that after excluding patients with antithyroid antibodies, goiter, and family history of thyroid disease, the mean TSH is 1.5 mIU/L; when this is extrapolated to be the Gaussian curve, the upper limit for the 97.5th percentile is actually 2.5 mIU/L [20].

### 2.3. Demographics, Drinking Profile, and Laboratory Evaluations

Data were collected for demographic indices (age, sex, body mass index [BMI]) and alcohol-drinking inventories (Table 1). Timeline Follow-back Instruments [22] and Lifetime Drinking History assessments [23] were employed to collect information on recent drinking history and chronic alcohol misuse, respectively. Drinking markers derived from these assessments included total drinks in the past 90 days (TD90), heavy drinking days in the past 90 days (HDD90), number of drinking days in the past 90 days (NDD90), and average drinks per day in the past 90 days (AvgD90). Further information on the collection of drinking history information is detailed in our previous publication [19]. All laboratory assays were analyzed at the Department of Laboratory Medicine of the National Institutes of Health, with a guideline set-up at the Medline Plus, which was set until 2014, when samples were assayed.

### 2.4. Laboratory Analyses

Serum samples were collected at baseline, and the following panels and values were collected: complete metabolic panel (CMP), complete blood count (CBC), thyroid panel (triiodothyronine (T3), free thyroxine (fT4), TSH, inflammatory markers (c reactive protein (CRP) and immunoglobulin A (IgA), G (IgG), and M (IgM)). Multiplex kits (Millipore, Billerica, MA, USA) on the Luminex (Luminex, Austin, TX, USA) platform were utilized for cytokine assays (TNF-α, IL-6, IL-8, IL-1β, PAI-1, MCP-1). LPS, LBP, and sCD14 were tested using Kinetic Chromogenic Limulus Amoebocyte Lysate Assay (Lonza, Walkersville, MD, USA), per manufacturer recommendations.

### 2.5. Statistical Analyses

SPSS v 28.01 (IBM, Chicago, IL, USA) and Microsoft 365 Excel (MS Corp, Redmond, WA, USA) were utilized for statistical analysis. Independent samples ANOVA was utilized to compare Gr.1 and Gr.2 overall by sex, analyzing demographics, drinking history, liver injury markers, thyroid function markers, blood cell measures, inflammatory markers, pro-inflammatory cytokines, and gut dysfunction markers. Univariate and multivariate regression analyses, further stratified by sex, were conducted to correlate drinking history with gut dysfunction markers and pro-inflammatory cytokines, cytokines with thyroid function markers, and cytokines with gut dysfunction. Receiver operating characteristic (ROC) curves were developed to determine the sensitivity and specificity of the cytokines and gut dysfunction markers in predicting thyroid function in the elevated TSH group (Gr.2) when compared to Gr.1. A *p*-value < 0.05 was used as a reference point to establish statistical significance. Data were presented as means with standard deviation (Mean ± SD) unless otherwise noted.

## 3. Results

### 3.1. Demographics and Drinking Profile

Gr.2 (elevated TSH) patients were older by approximately 5 years than patients in Gr.1 (normal TSH), though this was not statistically significant (Table 1). Both Gr.1 and Gr.2 had a borderline overweight BMI; females in Gr.2 were significantly more overweight than Gr.1 females, while Gr.1 females had a significantly higher BMI than males (Table 1). All measures of chronic and recent drinking history were elevated in Gr.2 compared to Gr.1, with statistically significant unique sex differences within and between the subgroups (Table 1).

### 3.2. Gut Dysfunction Markers and Pro-Inflammatory Responses

**Figure 1:**
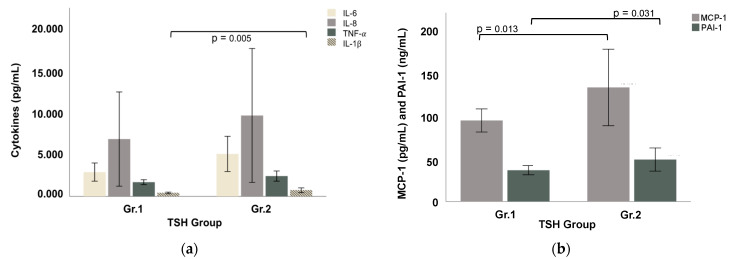
Proinflammatory cytokine levels in Gr.1 vs. Gr.2 patients. (**a**) IL-6, IL-8, TNF-α, IL-1β, and (**b**) MCP-1 and PAI-1. Significant differences between groups are denoted by *p*-values and seen in IL-1β, MCP-1, and PAI-1.

All pro-inflammatory cytokine levels were higher in Gr.2 than in Gr.1 (Table 1). In Gr.2, IL-6 was almost increased by 2-fold, while both IL-8 and TNF-α were increased by close to 1.5-fold compared to Gr.1 (Figure 1a). IL-1β was significantly higher in Gr.2, as well as uniquely higher among the females of Gr.2 (Table 1). MCP-1 and PAI-1 were both significantly higher in Gr.2 compared to Gr.1 (Figure 1b), both in overall patients as well as in males (Table 1). LPS was unchanged between the two groups. LBP and sCD14 were numerically higher in Gr.2 and showed significant sex differences within and between the subgroups (Table 1).

### 3.3. Thyroid Function and Non-Specific Inflammatory Markers

TSH was significantly higher, by more than two-fold, in Gr.2 compared to Gr.1, *p* = 0.046 (Table 1). The fT4 levels were above normal levels in both groups and significantly higher in Gr.2 compared to Gr.1. T3 was within normal limits in both groups and higher, by approximately 30 ng/dL, in Gr.2, though this was not statistically significant.

In Gr.2, patients had elevated AMCs, while they had lower ANCs, neither of which was statistically significant (Table 1). CRP was significantly higher, by approximately 2.5-fold, in Gr.2, *p* = 0.039. IgA, IgG, and IgM levels were minimally varied between the two groups, with insignificant differences.

### 3.4. Association of Drinking Markers and Measures of Gut Dysfunction and Pro-Inflammatory Status

In all the AUD patients (Gr.1 and Gr.2), HDD90 and NDD90 were significantly and independently associated with both LPS and LBP. TD90 was significantly associated with LBP. Correspondingly, unique significant relationships were found within Gr.2 patients. HDD90 and NDD90 were significantly associated with LBP, with *p* = 0.012 and *p* = 0.025, respectively. TD90 also significantly correlated with sCD14 within Gr.2, *p* = 0.038. In multivariate analyses, the same regression model yielded higher effects and significance in Gr.2; HDD90 demonstrated a significant association with LBP and sCD14 combined (r = 0.658, *p* = 0.033).

Alcohol-drinking markers also presented significant positive relationships with proinflammatory cytokines. TNF-α, one of the earliest cytokines in the pro-inflammatory cascade associated with AUD-related gut dysfunction [21], was significantly associated with NDD90 (*p* = 0.018) in Gr.2 patients and close to significance with HDD90 (*p* = 0.050). Among all groups, TNF-α was similarly significantly correlated with elevated NDD90 and HDD90. Further downstream in the inflammatory cascade, IL-6, IL-8, and IL-1β also significantly increased with alcohol use. In both groups combined, IL-6 was significantly correlated with NDD90, while IL-8 correlated significantly with TD90. Within both groups, and in Gr.2 specifically, IL-1β correlated significantly with TD90 and AvgDPD90.

Multivariate regression demonstrated several significant relationships between alcohol-drinking markers, gut dysfunction, and cytokine production (Table 2). In Gr.2, TD90 correlated significantly with IL-1β in the setting of the gut dysfunction markers LBP and sCD14. NDD90 correlated significantly with several cytokines in the setting of elevated LBP and sCD14, and with IL-6 (r = 0.736, *p* = 0.043), IL-1β (r = 0.741, *p* = 0.040), and PAI-1 (r = 0.786, *p* = 0.018) all within Gr.2 (Table 2). HDD demonstrated similarly significant associations in Gr.2 with the cytokines IL-6, IL-8, MCP-1, TNF-α, IL-1β, and PAI-1 when combined with LBP and sCD14 (Table 2). 

### 3.5. Characterization of Thyroid Dysregulation by the Gut–Immune–Brain and Gut–Thyroid Axes

**Figure 2:**
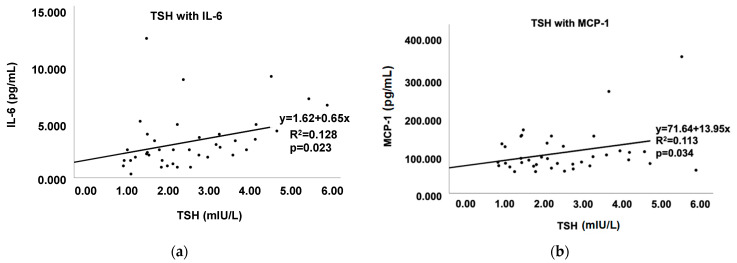
Correlation of TSH with pro-inflammatory cytokines among both Gr.1 and Gr.2 combined. (**a**) correlation between TSH and IL-6 and (**b**) correlation between TSH and MCP-1.

**Figure 3:**
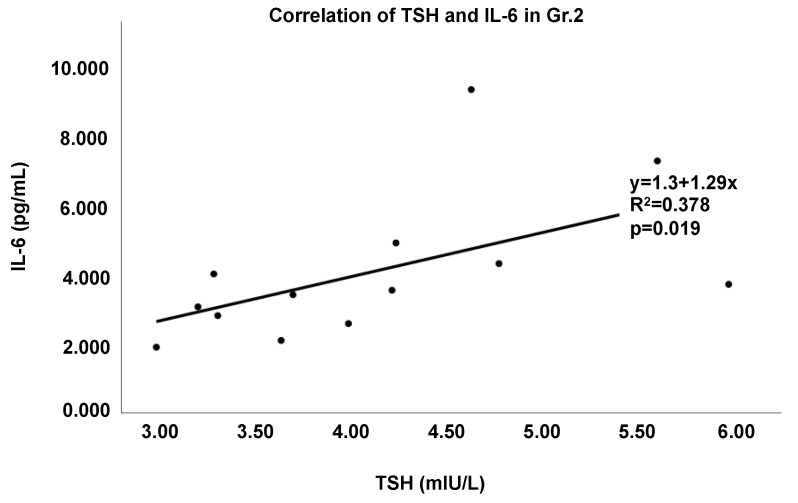
Regression analysis demonstrates the correlation between TSH and IL-6 in Gr.2 (elevated TSH). The r^2^ values and *p*-values are denoted.

Several pro-inflammatory cytokines were significantly associated with thyroid function among both groups of patients combined. TSH levels significantly correlated with IL-6 (Figure 2a), MCP-1 (Figure 2b), and TNF-α in both groups. Within Gr.2 particularly, TSH was significantly correlated with IL-6 (r = 0.615, *p* = 0.019) (Figure 3).

When combined with gut dysfunction markers in multivariate analysis, thyroid function demonstrated greater correlations with pro-inflammatory cytokines (Table 3). Within Gr.2 patients, in the setting of gut dysfunction (elevated LPS and LBP), TSH was significantly correlated with IL-6 and PAI-1 individually (Table 3). T3 and fT4 also demonstrated an increased correlation with PAI-1 (Table 3). Thyroid function also correlated significantly with several cytokines when analyzed with LBP and sCD14 combined; in particular, PAI-1 had strong correlations with TSH (r = 0.896, *p* = 0.002), T3 (r = 0.914, *p* = 0.004), and fT4 (r = 0.903, *p* = 0.006) (Table 3). These significant correlations were not seen in Gr.1 patients. Similarly, with all the gut dysfunction markers combined—LBP, sCD14, and LPS—thyroid function correlated significantly with PAI-1 in particular; this significance was seen with TSH (r = 0.905, *p* = 0.005), T3 (r = 0.919, *p* = 0.013), and fT4 (r = 0.906, *p* = 0.020) (Table 3).

### 3.6. Assessment of Alcohol and Its Effect on the Gut–Brain–Thyroid Axis

When analyzing the effect of alcohol-induced gut dysfunction and pro-inflammatory state on thyroid function, there were highly significant correlations demonstrated within Gr.2 in particular. Thyroid function demonstrated significant correlations with TD90, the gut dysfunction markers sCD14 and LBP, and the pro-inflammatory cytokine IL-6 (r = 0.893, *p* = 0.007). A similarly strong relationship was seen with PAI-1 (r = 0.914, *p* = 0.003). Thyroid function also correlated significantly with NDD90, sCD14, LBP, and with multiple pro-inflammatory cytokines; this includes IL-6 (r = 0.902, *p* = 0.005), IL-8 (r = 0.887, *p* = 0.009), MCP-1 (r = 0.926, *p* = 0.002), TNF-α (r = 0.911, *p* = 0.004), IL-1β (r = 0.894, *p* = 0.007), and PAI-1 (r = 0.905, *p* = 0.005). Thyroid function had similarly significant correlations with HDD and with all of the above pro-inflammatory cytokines in the setting of gut dysfunction; this includes IL-6 (r = 0.891, *p* = 0.007), IL-8 (r = 0.885, *p* = 0.009), MCP-1 (r = 0.922, *p* = 0.002), TNF-α (r = 0.907, *p* = 0.004), IL-1β (r = 0.888, *p* = 0.008), and PAI-1 (r = 0.893, *p* = 0.007). 

### 3.7. Diagnostic Assessment of Inflammation and Gut Dysfunction in Thyroid Function

**Figure 4:**
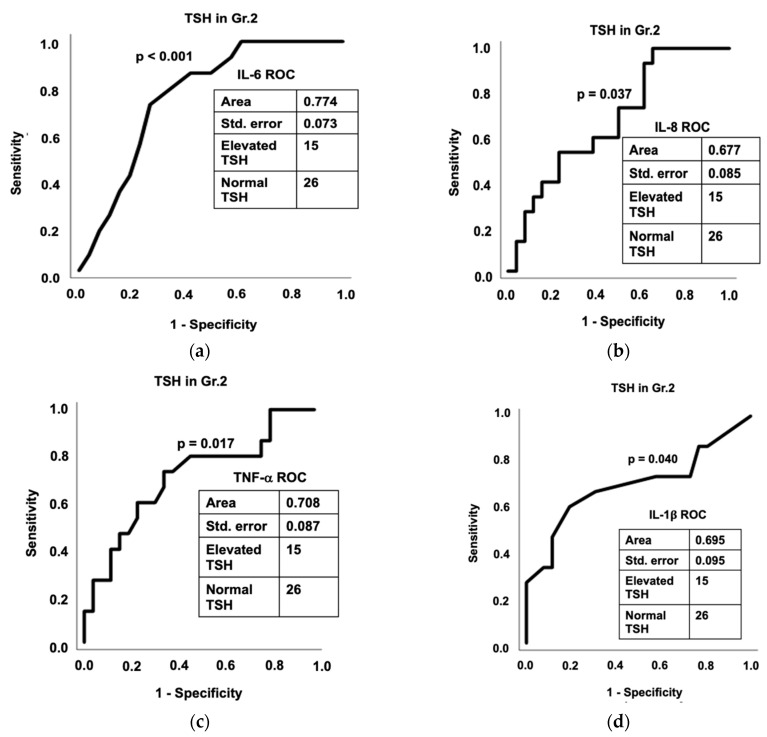

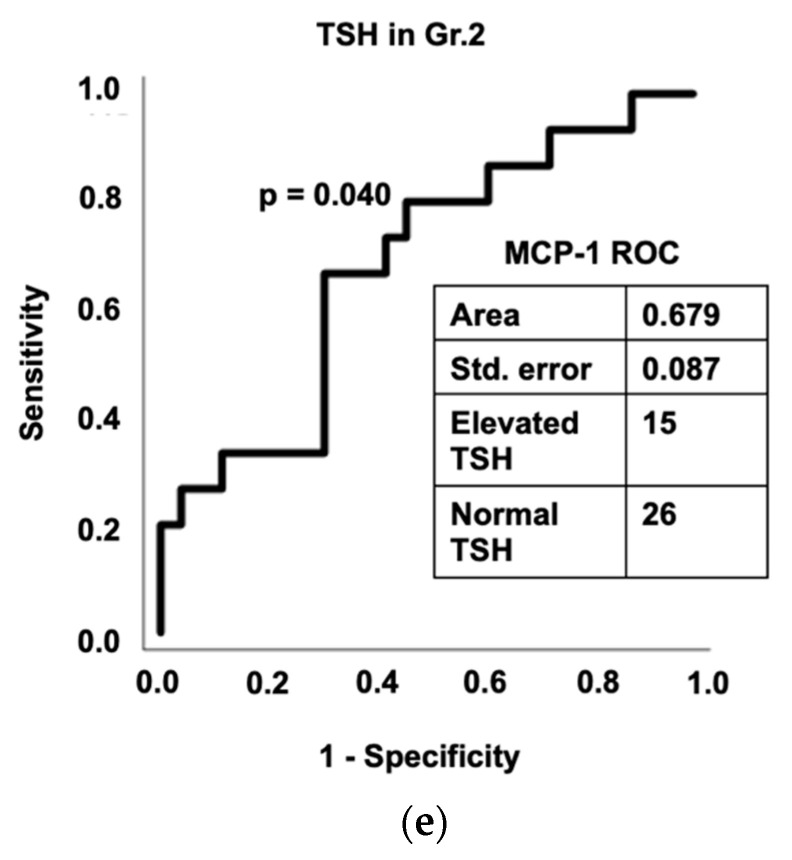
AUROC analysis demonstrating the diagnostic ability of several of the cytokines to predict TSH levels within Gr.2: (**a**) IL-6; (**b**) IL-8; (**c**) TNF-α; (**d**) IL-1β; and (**e**) MCP-1. Area under the curve, standard error, number of patients within Gr.2 with elevated TSH, and number of patients within Gr. 1 with normal TSH are reported for each analysis.

Within Gr.2, several inflammatory cytokines had high predictive efficacy for hypothyroidism caused by elevated TSH. Within Gr.2, TNF-α significantly predicted an elevated TSH, area 0.708, *p* = 0.017 (Figure 4c). Further downstream in the inflammatory pathway, IL-6, IL-8, IL-1β, and MCP-1 significantly predicted elevated TSH (Figure 4a,b,d,e). PAI-1 also had high predictive efficacy, though this was not statistically 

## 4. Discussion

Our study examined the effects of heavy and chronic alcohol use on thyroid function by way of alcohol-induced gut dysfunction and pro-inflammatory cytokine release. Overall, the study patients did not have large differences in age or BMI. Gr.2 patients drank more chronically, as determined by LTDH, more frequently, as determined by TD90 and NDD90, and more heavily, as determined by HDD90 and AvgDPD90. Correspondingly, this group demonstrated higher gut dysfunction markers and pro-inflammatory cytokine levels. Alcohol has long been known to play a critical role in the activation of the innate and adaptive immune systems. There are several studies that have described the correlation between alcohol consumption and the associated immune responses. Chronic alcohol use, in particular, is associated with an upregulation of NF-kB activation by LPS; this leads to increased activation of monocytes and macrophages, instigating a large surge in the release of pro-inflammatory cytokines, such as TNF-α, IL-6, and IL-8 [24,25,26]. Specifically, chronic alcohol use results in microbial proliferation and the acetaldehyde-mediated opening of intestinal tight junctions, which results in increased endotoxin release into the circulation [27,28,29]. The endotoxin LPS, with the cooperation of LBP and co-receptor sCD14, binds to TLR4 in the liver and gut; this activates signaling cascades which upregulate NF-kB and eventually increase the production of pro-inflammatory cytokines [30]. In addition to gut dysfunction-induced cytokine production, alcohol has also been shown to directly activate inflammasomes in multiple tissues after chronic use [31,32]; this results in the induction of caspase-1, which is required to form the mature forms of IL-1β, among other cytokines [33,34]. This alcohol-induced dysfunction was similarly demonstrated in our study.

In this study, all of the patients enrolled had baseline AUD with a history of chronic and acute drinking habits. However, it was particularly the acute drinking history, especially HDD90, TD90, and NDD990, which had the strongest correlation with gut dysfunction and pro-inflammatory cytokine responses. This could suggest that in the setting of chronic alcohol abuse, the increase in acute alcohol intake acts as a second hit, further worsening the gut dysfunction and cytokine surges. NDD90 and HDD90 had moderately strong and significant correlations with gut dysfunction markers in Gr.2, particularly with LBP and sCD14 combined; TD90, NDD90, and HDD had moderately strong correlations with several cytokines, such as IL-6, IL-8, IL-1β, and TNF-α as well. When adding cytokines to the alcohol–gut relationship, there was a large increase in the strength of correlation. This coincides well with the understanding that alcohol-induced gut dysfunction contributes to the alcohol-induced pro-inflammatory state via the action of endotoxins on toll-like receptors. 

In our study, the thyroid response (TSH, T3, and fT4) to the gut–cytokine relationship indicates a positive response, particularly with cytokines further downstream in the inflammatory cascade, such as PAI-1 and IL-6. In addition, several inflammatory cytokines also significantly predicted TSH levels within Gr.2, which was not seen in Gr.1. This further contributes to the hypothesis that alcohol-induced gut dysfunction and the pro-inflammatory cytokine response have an effect on thyroid function. There are studies that have previously demonstrated relationships between pro-inflammatory cytokines and thyroid function. Several studies demonstrate that IL-6 and IL-8 levels are significantly higher in patients with subacute thyroiditis when compared to normal healthy controls [35,36,37]. This relationship between thyroid hormones and the immune system is thought to be bidirectional or constitute part of a feedback loop. Some studies elucidate an association between thyroidectomy, hypothyroidism, and thymic growth depression with decreased circulating lymphocytes [38,39,40,41]. Furthermore, Blalock et al. initially demonstrated that TSH induced an increase in the immune response by increasing the production of MCP-1 and IL-6 [42]. Conversely, other studies reveal that acute infections indirectly increase thyroid hormone release via the action of IL-1, IL-6, and TNF-α on the hypothalamus, decreasing TSH release from the pituitary. There is clearly a mutual relationship between these inflammatory molecules and thyroid hormones, and our study furthers this understanding due to the statistically significant correlations observed between gut dysfunction, cytokine production, and thyroid dysfunction. 

Interestingly, Gr.2 also had higher T3, albeit within normal levels, and higher fT4 levels; this is discordant with the classic hypothyroid picture expected with elevated TSH. In fact, when we examined this further, every patient in Gr.2 had an above normal fT4; this indicates that there are multiple factors modulating thyroid function, likely both at the glandular level as well as in the central nervous system (CNS). The relationship between alcohol use and the HPT axis has previously been investigated, though there is no consensus on the exact effects of alcohol use on the thyroid or the underlying pathogenesis. Some studies have demonstrated a blunted or absent response of TSH to TRH in patients with a history of alcohol abuse [43,44]. Other studies have further explored this effect and determined that in these patients, there were decreased concentrations of TSH at baseline and after stimulation with TRH [45], which insinuates alcohol has some effect on the CNS, likely on the pituitary gland. Several authors have also suggested that pituitary-TRH receptors are downregulated due to chronically high TRH concentrations in chronic alcohol use, as demonstrated by human and rat models [46,47]. Other studies have also examined the effect of alcoholism on peripheral thyroid hormones. Hegedus et al. demonstrated a significant reduction in the thyroid gland volume in alcohol-dependent patients in multiple studies [48,49]. Ultrasound images of the gland demonstrated not only a decrease in volume of the gland but also a higher level of fibrosis with even a short duration of excessive alcohol use; the authors suggested that this may be due to a direct effect of alcohol on the gland itself [50,51]

**Figure 5:**
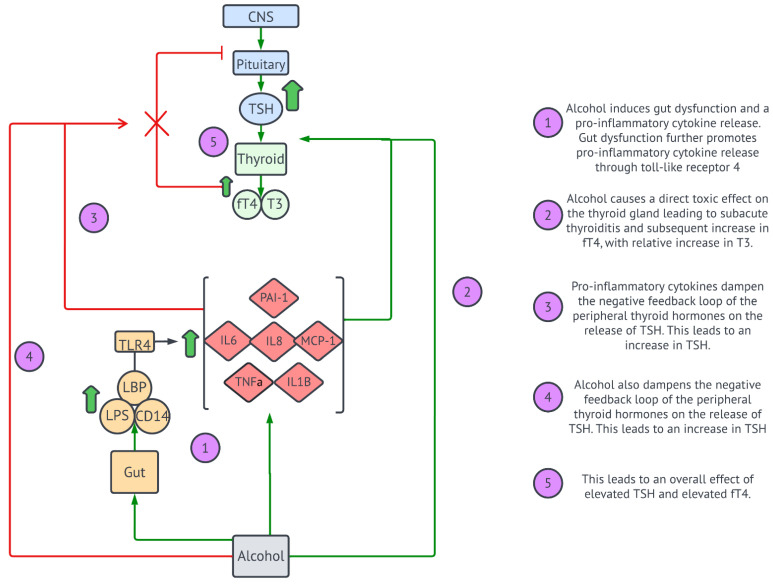
Our study hypothesis on the effects of alcohol on the HPT axis. This demonstrates (1) the pathogenesis of alcohol-induced gut dysfunction and pro-inflammatory responses; (2) the direct effect of alcohol on the thyroid; (3) the effect of pro-inflammatory cytokines on the HPT axis; (4) the effect of alcohol on the HPT axis; and (5) the resulting presentation of elevated TSH and elevated fT4.

Our study postulated the effects of alcohol, gut dysfunction, and pro-inflammatory cytokines on the HPT axis (Figure 5). Interestingly, Gr.2 patients had elevated TSH, as well as elevated fT4, while T3 was within normal limits. One possibility is that alcohol abuse results in a direct toxic effect on the thyroid gland, resulting in its inflammation and leading to subacute thyroiditis; this would account for the elevated fT4 in both groups, which was even higher in Gr.2. The likelihood of elevated fT4 being secondary to subacute thyroiditis is strengthened by the significantly elevated CRP levels in Gr.2. In fact, several studies have demonstrated a strong correlation between thyroid function and elevated CRP levels in patients primarily with subacute thyroiditis; this correlation is not necessarily seen in other inflammatory thyroid conditions [52,53,54]. T3 was normal in both groups, which indicates that even with elevated fT4 levels, there was decreased iodation. This is consistent with several studies that establish that in the case of elevated inflammatory cytokines, such as IL-6, or systemic illness, there is decreased conversion of T4 into T3 peripherally, regardless of TSH levels [55,56,57]. With elevated fT4 levels, TSH levels are expected to be low or normal due to the negative feedback of peripheral thyroid hormones on the pituitary and the hypothalamus [58]. However, in our study, every patient with elevated TSH had an elevated fT4, suggesting there was a disruption of this negative feedback loop; we hypothesize that this could be either at the hypothalamus and/or the pituitary level (Figure 5). Interestingly, this strengthens prior hypotheses that alcohol may not only have toxic effects on the thyroid gland but also, more centrally, on the HPT axis. This is a novel finding in a small population size and would be an interesting relationship to explore in larger studies with well-structured study designs and focused hypotheses. 

There are several limitations to this study. Part of the extent of the limitations lies in the scope of the study. This is a novel finding and was conducted as a clinical-translational paradigm. Thus, the aims were very specific and did not include the offshoots or special sub-categorial group studies. The sample size was small to moderate, and this was not a large-scale study. Thus, many underlying effects that were identified during the course of data evaluation were not investigated. Several findings from this novel study create interest in addressing other scientific goals. These may need different study designs independent of this project and on a larger scale with a well-defined hypothesis. The study was also based on a single timepoint, so the thyroid function over a period of time was not evaluated. There was no TRH collected for any of the patients, which would have provided even more of an understanding of the mechanism of CNS dysfunction. In addition, we did not collect other pituitary-induced hormones, such as cortisol. However, the likelihood of AUD causing significant changes in the adrenocorticotropin hormone (ACTH)–cortisol pathway is low, considering that it would likely require significant disease. These patients were also admitted for treatment for acute alcohol intoxication, but there was no control of the level of intoxication or whether the patients had already started the withdrawal process. The different levels of stress associated with these stages may have affected thyroid levels. Future studies can aim to address these limitations when investigating these relationships.

As this is a pilot study examining a largely uninvestigated relationship between alcohol use and thyroid function, there are still several components that will need to be expanded on in future studies. We focused on the functional and pathophysiological pathways involved with alcohol-induced gut inflammation leading to altered thyroid function. However, further examination into the particular microbiota that may be involved in this pathophysiology might contribute to a further understanding of these pathways. Gut dysfunction markers in this study were limited to LPS, LBP, and sCD14, which we demonstrate have a significant association with gut-dysfunction-associated cytokines, and we conclude that they are important markers of gut dysfunction. Other markers, such as fecal calprotectin or plasma citrulline, may also complement these primary markers in determining gut dysfunction. These can and should be evaluated in future studies.

While there are several studies demonstrating the effects of pro-inflammatory cytokines and the direct effects of alcohol on the HPT axis on an individual basis, our study was the first of its kind to describe alcohol-induced gut dysfunction and pro-inflammatory cytokine response in thyroid function. There is a high chance that in the AUD patients enrolled in our study, the HPT axis demonstrated dysregulation at several levels (Figure 5). This includes the direct effects on the thyroid gland to produce subacute thyroiditis. These, combined with the effects centrally, dampen the negative feedback loop of the elevated peripheral thyroid hormones. This is a novel finding of alcohol-induced thyroid dysfunction that has only begun to determine the unique underlying pathogenesis. This study dictates the need for further study of this unique correlation, as well as the long-term effects on the thyroid after the discontinuation of alcohol drinking. 

## Figures and Tables

**Table 1:** At baseline—demographic, drinking history, liver injury measures, nutritional status, candidate blood panel measures, cytokines, and gut dysfunction markers of alcohol use disorder of patients tabulated by normal versus elevated TSH levels.

	Group 1 (Normal TSH, Gr. 1)			Group 2 (Elevated TSH, Gr. 2)			Between Group *p*–Value
	Males (*n* = 21; 75%)	Females (*n* = 7; 25%)	Total (*n* = 28; 63.6%)	Males (*n* = 10; 62.5%)	Females (*n* = 6; 37.5%)	Total (*n* = 16; 36.4%)	
**Demographics**							
**Age ^c^ (years)**	41.6 ± 8.5	41.3 ± 13.8	41.5 ± 9.8	47.3 ± 12.1	44.7 ± 11.6	46.3 ± 11.6	ns
**BMI ^bc^ (kg/m^2^)**	26.3 ± 4.4	29.5 ± 10.5	27.1 ± 6.5	27.8 ± 4.6	26.3 ± 2.7	27.3 ± 4.0	ns
**Drinking History**							
**TD90**	1091 ± 450	915 ± 664	1052 ± 496	1159 ± 686	1084 ± 604	1131 ± 637	ns
**HDD90**	71.43 ± 22.02	61.33 ± 21.26	69.19 ± 21.87	73.60 ± 21.58	75.83 ± 18.44	74.44 ± 19.85	ns
**AvgDPD90 ^ac^**	14.32 ± 3.89	14.91 ± 9.26	14.45 ± 5.31	15.68 ± 7.65	13.57 ± 6.10	14.89 ± 6.97	ns
**NDD90**	75.14 ± 18.26	62.50 ± 20.93	72.33 ± 19.22	75.50 ± 22.48	77.67 ± 16.07	76.31 ± 19.76	ns
**LTDH**	15.80 ± 9.60	9.86 ± 5.21	14.26 ± 8.98	21.90 ± 9.69	11.83 ± 9.00	18.13 ± 10.42	ns
**Liver Panel**							
**ALT (IU/L)**	81.86 ± 40.93	58.57 ± 77.88	76.04 ± 51.90	80.00 ± 41.00	94.50 ± 108.70	85.44 ± 70.71	ns
**AST (IU/L)**	92.57 ± 72.12	88.14 ± 134.12	91.46 ± 88.62	114.40 ± 103.13	158.33 ± 122.50	130.88 ± 108.93	ns
**AST:ALT**	1.091 ± 0.553	1.408 ± 0.459	1.170 ± 0.541	1.300 ± 0.626	1.970 ± 0.937	1.551 ± 0.800	ns
**TBili (mg/dL)**	0.786 ± 0.869	0.743 ± 0.365	0.775 ± 0.767	0.690 ± 0.247	0.733 ± 0.398	0.706 ± 0.300	ns
**Albumin (g/dL)**	4.181 ± 0.423	4.029 ± 0.206	4.143 ± 0.382	4.080 ± 0.358	4.217 ± 0.458	4.131 ± 0.389	ns
**Thyroid Function**							
**TSH (mIU/L)**	1.781 ± 0.609	1.793 ± 0.711	1.784 ± 0.622	3.931 ± 0.951	4.656 ± 1.001	4.173 ± 0.996	0.046
**T3 (ng/dL)**	106.45 ± 19.22	109.33 ± 30.12	107.47 ± 22.72	131.88 ± 20.32	146.25 ± 17.75	136.67 ± 19.97	ns
**fT4 (ng/dL)**	5.782 ± 1.154	5.700 ± 1.101	5.753 ± 1.101	5.575 ± 1.456	7.950 ± 0.507	6.367 ± 1.669	0.037
**Nutritional Status**							
**Zinc (ug/dL)**	79.53 ± 12.19	64.43 ± 12.30	75.46 ± 13.78	71.90 ± 13.37	92.67 ± 46.26	79.69 ± 30.47	ns
**Blood Cell Measures**							
**AMC ^c^ (K/uL)**	0.492 ± 0.233	0.489 ± 0.126	0.491 ± 0.209	0.535 ± 0.268	0.518 ± 0.076	0.528 ± 0.212	ns
**ANC (K/uL)**	3.684 ± 2.063	5.216 ± 1.872	4.067 ± 2.095	3.266 ± 1.799	3.370 ± 1.280	3.305 ± 1.578	ns
**Inflammatory markers**							
**CRP ^c^ (mg/L)**	2.07 ± 1.61	0.57 ± 0.04	1.42 ± 1.39	4.47 ± 7.16	0.88 **	3.75 ± 6.40	0.039
**IgA (pg/mL)**	295.9 ± 113.7	268.3 ± 128.4	289.0 ± 115.7	281.1 ± 173.7	249.7 ± 86.0	269.3 ± 144.3	ns
**IgG (pg/mL)**	1102 ± 439	1152 ± 213	1114 ± 391	1169 ± 314	1010 ± 254	1109 ± 295	ns
**IgM ^d^ (pg/mL)**	123.4 ± 63.5	106.9 ± 48.7	119.3 ± 59.7	113.8 ± 41.6	156.8 ± 90.2	129.9 ± 64.9	ns
**Candidate Cytokine Response**							
**IL-6 (pg/mL)**	2.588 ± 2.537	3.771 ± 3.101	2.861 ± 2.659	4.268 ± 2.250	6.102 ± 5.357	5.002 ± 3.743	ns
**IL-8 ^cd^ (pg/mL)**	4.013 ± 3.247	15.886 ± 27.849	6.753 ± 13.753	5.581 ± 2.410	15.463 + 22.137	9.534 ± 14.263	ns
**TNF-α ^d^ (pg/mL)**	1.743 ± 0.693	1.458 ± 0.779	1.677 ± 0.708	2.215 ± 0.562	2.659 ± 1.624	2.393 ± 1.083	ns
**IL-1β ^b^ (pg/mL)**	0.451 ± 0.227	0.268 ± 0.196	0.409 ± 0.230	0.688 ± 0.378	0.772 ± 0.689	0.722 ± 0.503	0.005
**MCP-1 ^a^ (pg/mL)**	96.62 ± 32.57	91.47 ± 39.87	95.43 ± 33.60	123.92 ± 63.64	149.90 ± 107.31	134.31 ± 81.24	0.013
**PAI-1 ^a^ (ng/mL)**	35.91 ± 13.80	40.36 ± 11.98	36.94 ± 13.31	56.86 ± 24.65	38.34 ± 21.69	49.46 ± 24.56	0.031
**Candidate Gut Dysfunction Markers**							
**LPS (EU/mL)**	0.106 ± 0.060	0.091 ± 0.058	0.102 ± 0.059	0.097 ± 0.064	0.109 ± 0.069	0.102 ± 0.064	ns
**LBP ^abcd^ (ng/mL)**	1119 ± 1430	3989 ± 4989	1781 ± 2838	2822 ± 3480	751 ± 624	2132 ± 2986	ns
**sCD14 ^d^ (ng/mL)**	9063 ± 1970	9375 ± 1323	9141 ± 1812	8554 ± 1840	11321 ± 733	9592 ± 2031	ns

Between-group *p*-values compare totals between Gr.1 and Gr.2. Significant between-group analyses were also conducted for: ^a^ males only between Gr. 1 and Gr. 2, ^b^ females only between Gr.1 and Gr.2, ^c^ between sex in Gr. 1 only, and ^d^ between sex in Gr. 2 only. ns: not significant; BMI: Body mass index; TD90: Total drinks past 90 days; HDD90: heavy drinking days past 90 days; AvgDPD90: Average drinks per drinking day past 90 days; NDD90: number of drinking days past 90 days; NNDD90: number of non-drinking days past 90 days, LTDH: lifetime drinking history (in years), ALT: serum alanine aminotransferase, AST: serum aspartate aminotransferase, AST:ALT: ratio of AST by ALT, Tbili: Total bilirubin, TSH: thyroid-stimulating hormone, T3: triiodothyronine, fT4: free thyroxine, WBC: white blood cells count, AMC: absolute monocyte count, ANC: absolute neutrophil count, CRP: c reactive protein, IgA: immunoglobulin A, IgG: immunoglobulin G, IgM: immunoglobulin M, Ω6/Ω3: omega-6 to omega-3 ratio, IL-6: interleukin 6, IL-8: interleukin 8, TNF-α: tumor-like necrotic factor alpha, Il-1β: interleukin 1 beta, MCP-1: monocyte chemoattractant protein-1, PAI-1: plasminogen activator inhibitor-1, LPS: lipopolysaccharide, LBP: LPS binding protein, sCD14: LPS-induced pathogen-associated protein. ** There was only one data point for this particular category.

**Table 2:** Multivariate regression analysis between drinking markers, gut dysfunction markers, and pro-inflammatory cytokine response. R values are reported, along with the associated *p*-value, if significant.

	LBP + sCD14												LPS + LBP + sCD14	
	TNF-α		IL-6		IL-8		IL-1β		MCP-1		PAI-1		IL-1β	
**TD90**	ns	ns	ns	ns	ns	ns	0.827	*p* = 0.007	ns	ns	ns	ns	0.827	*p* = 0.023
**NDD90**	ns	ns	0.736	*p* = 0.043	ns	ns	0.741	*p* = 0.040	ns	ns	0.786	*p* = 0.018	ns	ns
**HDD**	0.759	*p* = 0.030	0.788	*p* = 0.018	0.769	*p* = 0.025	0.774	*p* = 0.023	0.757	*p* = 0.031	0.798	*p* = 0.014	ns	ns

ns: not significant; TD90: Total drinks past 90 days; NDD90: number of drinking days past 90 days; HDD90: heavy drinking days past 90 days; TNF-α: tumor necrosis factor-α; IL-6: interleukin 6; IL-8: interleukin 8; IL-1β: interleukin 1β; MCP-1: monocyte chemoattractant protein-1; PAI-1: plasminogen activator inhibitor-1; LPS: lipopolysaccharide; LBP: LPS binding protein; sCD14: LPS-induced pathogen-associated protein.

**Table 3:** A multivariate regression analysis of gut dysfunction markers, pro-inflammatory cytokine response, and thyroid function. R values are reported, along with the associated *p*-value, if significant.

	LPS + LBP				LBP + sCD14				LPS + LBP + sCD14			
	IL-6		PAI-1		IL-6		PAI-1		IL-6		PAI-1	
**TSH**	0.756	*p* = 0.046	0.800	*p* = 0.022	0.816	*p* = 0.016	0.896	*p* = 0.002	0.819	*p* = 0.043	0.905	*p* = 0.005
**T3**	ns	ns	0.878	*p* = 0.012	ns	ns	0.914	*p* = 0.004	ns	ns	0.919	*p* = 0.013
**fT4**	ns	ns	0.854	*p* = 0.021	ns	ns	0.903	*p* = 0.006	ns	ns	0.906	*p* = 0.020

ns: not significant. IL-6: interleukin 6; PAI-1: plasminogen activator inhibitor-1; LPS: lipopolysaccharide; LBP: LPS binding protein; sCD14: LPS-induced pathogen-associated protein; TSH: thyroid-stimulating hormone; T3: triiodothyronine; fT4: free thyroxine.

## Data Availability

The datasets analyzed during the current study are available from the corresponding author upon reasonable request. Email address for the readers to contact the author to obtain the data: v0vats01@louisville.edu.

## References

[1] Tucker J.A., Chandler S.D., Witkiewitz K. (2020). Epidemiology of recovery from alcohol use disorder. Alcohol Res. Curr. Rev..

[2] (2021). Alcohol Use in the United States National Institute on Alcohol Abuse and Alcoholism. https://www.niaaa.nih.gov/publications/brochures-and-fact-sheets/alcohol-facts-and-statistics#:~:text=Prevalence%20of%20Drinking%3A%20According%20to,in%20this%20age%20group%20and.

[3] Keyes K.M. (2022). Age, period, and cohort effects in alcohol use in the United States in the 20th and 21st centuries: Implications for the coming decades. Alcohol Res. Curr. Rev..

[4] Goldberg M. (1962). Thyroid function in chronic alcoholism. Lancet.

[5] Zoeller R.T., Fletcher D.L., Simonyl A., Rudeen P.K. (1996). Chronic ethanol treatment reduces the responsiveness of the hypothalamic-pituitary-thyroid axis to central stimulation. Alcohol. Clin. Exp. Res..

[6] Loosen P.T., Wilson I.C., Dew B.W., Tipermas A. (1983). Thyrotropin-releasing hormone (TRH) in abstinent alcoholic men. Am. J. Psychiatry.

[7] Casacchia M., Rossi A., Stratta P. (1985). Thyrotropin-releasing hormone test in recently abstinent alcoholics. Psychiatry Res..

[8] Ozsoy S., Esel E., Izgi H.B., Sofuoglu S. (2006). Thyroid function in early and late alcohol withdrawal: Relationship with aggression, family history, and onset age of alcoholism. Alcohol Alcohol..

[9] Hermann D., Heinz A., Mann K. (2002). Dysregulation of the hypothalamic-pituitary-thyroid axis in alcoholism. Addiction.

[10] Canesso M.C.C., Lacerda N.L., Ferreira C.M., Goncalves J.L., Almeida D., Gamba C., Cassali G., Pedroso S.H., Moreira C., Martins F.S. (2014). Comparing the effects of acute alcohol consumption in germ-free and conventional mice: The role of the gut microbiota. BMC Microbiol..

[11] Leclercq S., De Saeger C., Delzenne N., de Timary P., Starkel P. (2014). Role of inflammatory pathways, blood mononuclear cells, and gut-derived bacterial products in alcohol dependence. Biol. Psychiatry.

[12] Bull-Otterson L., Feng W., Kirpich I., Wang Y., Qin X., Liu Y., Gobejishvili L., Joshi-Barve S., Ayvaz T., Petrosino J. (2013). Metagenomic analyses of alcohol induced pathogenic alterations in the intestinal microbiome and the effect of *Lactobacillus rhamnosus GG* treatment. PLoS ONE.

[13] Pijls K.E., Jonkers D.M., Elamin E.E., Masclee A.A., Koek G.H. (2013). Intestinal epithelial barrier function in liver cirrhosis: An extensive review of the literature. Liver Int..

[14] Malaguarnera G., Giordano M., Nunnari G., Bertino G., Malaguarnera M. (2014). Gut microbiota in alcoholic liver disease: Pathogenetic role and therapeutic perspectives. World J. Gastroenterol..

[15] Forsyth C.B., Voigt R.M., Keshavarzian A. (2014). Intestinal CYP2E1: A mediator of alcohol-induced gut leakiness. Redox Biol..

[16] Fleming S., Toratani S., Shea-Donohue T., Kashiwabara Y., Vogel S.N., Metcalf E.S. (2001). Pro- and anti-inflammatory gene expression in the murine small intestine and liver after chronic exposure to alcohol. Alcohol. Clin. Exp. Res..

[17] Uesugi T., Froh M., Arteel G.E., Bradford B.U., Thurman R.G. (2001). Toll-like receptor 4 is involved in the mechanism of early alcohol-induced liver injury in mice. Hepatology.

[18] Wang M., Ma L.J., Yang Y., Xiao Z., Wan J.B. (2019). n-3 Polyunsaturated fatty acids for the management of alcoholic liver disease: A critical review. Crit. Rev. Food Sci. Nutr..

[19] Vatsalya V., Song M., Schwandt M.L., Cave M.C., Barve S.S., George D.T., Ramchandani V.A., McClain C.J. (2016). Effects of sex, drinking history, and omega-3 and omega-6 fatty acids dysregulation on the onset of liver injury in very heavy drinking alcohol-dependent patients. Alcohol. Clin. Exp. Res..

[20] Wartofsky L., Dickey R.A. (2005). The evidence for a narrower thyrotropin reference range is compelling. J. Clin. Endocrinol. Metab..

[21] Spencer C.A., Hollowell J.G., Kazarosyan M., Braverman L.E. (2007). National health and nutrition examination survey III thyroid-stimulating hormone (TSH)-thyroperoxidase antibody relationships demonstrate that TSH upper reference limits may be skewed by occult thyroid dysfunction. J. Clin. Endocrinol. Metab..

[22] Sobell L.C., Sobell M.B. (1992). Timeline follow-back. Measuring Alcohol Consumption.

[23] Skinner H.A., Sheu W.-J. (1982). Reliability of alcohol use indices. The lifetime drinking history and the MAST. J. Stud. Alcohol.

[24] McClain C.J., Barve S., Deaciuc I., Kugelmas M., Hill D. (1999). Cytokines in alcoholic liver disease. Seminars in Liver Disease.

[25] Means T.K., Lien E., Yoshimura A., Wang S., Golenbock D.T., Fenton M.J. (1999). The CD14 ligands lipoarabinomannan and lipopolysaccharide differ in their requirement for Toll-like receptors. J. Immunol..

[26] Lien E., Sellati T.J., Yoshimura A., Flo T.H., Rawadi G., Finberg R.W., Carroll J.D., Espevik T., Ingalls R.R., Radolf J.D. (1999). Toll-like receptor 2 functions as a pattern recognition receptor for diverse bacterial products. J. Biol. Chem..

[27] Rao R.K., Seth A., Sheth P. (2004). Recent advances in alcoholic liver disease I. Role of intestinal permeability and endotoxemia in alcoholic liver disease. Am. J. Physiol.-Gastrointest. Liver Physiol..

[28] Yumuk Z., Ozdemirci S., Erden B.F., Dundar V. (2001). The effect of long-term ethanol feeding on *Brucella melitensis* infection of rats. Alcohol Alcohol..

[29] Kavanaugh M.J., Clark C., Goto M., Kovacs E.J., Gamelli R.L., Sayeed M.M., Choudhry M.A. (2005). Effect of acute alcohol ingestion prior to burn injury on intestinal bacterial growth and barrier function. Burns.

[30] Takeda K., Akira S. (2004). TLR signaling pathways. Semin. Immunol..

[31] Szabo G., Lippai D. (2014). Converging actions of alcohol on liver and brain immune signaling. Int. Rev. Neurobiol..

[32] Orman E.S., Odena G., Bataller R. (2013). Alcoholic liver disease: Pathogenesis, management, and novel targets for therapy. J. Gastroenterol. Hepatol..

[33] Alfonso-Loeches S., Pascual-Lucas M., Blanco A.M., Sanchez-Vera I., Guerri C. (2010). Pivotal role of TLR4 receptors in alcohol-induced neuroinflammation and brain damage. J. Neurosci..

[34] Tsuchiya K., Hara H. (2014). The inflammasome and its regulation. Crit. Rev. Immunol..

[35] Bartalena L., Brogioni S., Grasso L., Martino E. (1993). Increased serum interleukin-6 concentration in patients with subacute thyroiditis: Relationship with concomitant changes in serum T4-binding globulin concentration. J. Endocrinol. Investig..

[36] Siddiqi A., Monson J.P., Wood D.F., Besser G.M., Burrin J.M. (1999). Serum cytokines in thyrotoxicosis. J. Clin. Endocrinol. Metab..

[37] Kobawala T.P., Patel G.H., Gajjar D.R., Patel K.N., Thakor P.B., Parekh U.B., Patel K.M., Shukla S.N., Shah P.M. (2011). Clinical utility of serum interleukin-8 and interferon-alpha in thyroid diseases. J. Thyroid. Res..

[38] Fabris N., Pierpaoli W., Sorkin E. (1971). Hormones and the immunological capacity. IV. Restorative effects of developmental hormones or of lymphocytes on the immunodeficiency syndrome of the dwarf mouse. Clin. Exp. Immunol..

[39] Comsa J., Leonhardt H., Ozminski K. (1979). Hormonal influences on the secretion of the thymus. Thymus.

[40] King D.B., Scanes C.G. (1986). Effect of mammalian growth hormone and prolactin on the growth of hypophysectomized chickens. Proc. Soc. Exp. Biol. Med..

[41] De Vito P., Incerpi S., Pedersen J.Z., Luly P., Davis F.B., Davis P.J. (2011). Thyroid hormones as modulators of immune activities at the cellular level. Thyroid.

[42] Blalock J.E., Johnson H.M., Smith E.M., Torres B.A. (1984). Enhancement of the in vitro antibody response by thyrotropin. Biochem. Biophys. Res. Commun..

[43] Pienaar W.P., Roberts M.C., Emsley R.A., Aalbers C., Taljaard F.J. (1995). The thyrotropin releasing hormone stimulation test in alcoholism. Alcohol Alcohol..

[44] Sellman J.D., Joyce P.R. (1992). The clinical significance of the thyrotropin-releasing hormone test in alcoholic men. Aust. N. Z. J. Psychiatry.

[45] Valimaki M., Pelkonen R., Harkonen M., Ylikahri R. (1984). Hormonal changes in noncirrhotic male alcoholics during ethanol withdrawal. Alcohol Alcohol..

[46] Winokur A., Caroff S.N., Amsterdam J.D., Maislin G. (1984). Administration of thyrotropin-releasing hormone at weekly intervals results in a diminished thyrotropin response. Biol. Psychiatry.

[47] Sheppard M.C., Shennan K.I. (1984). Desensitization of rat anterior pituitary gland to thyrotrophin releasing hormone. J. Endocrinol..

[48] Hegedus L. (1984). Decreased thyroid gland volume in alcoholic cirrhosis of the liver. J. Clin. Endocrinol. Metab..

[49] Hegedus L., Rasmussen N., Ravn V., Kastrup J., Krogsgaard K., Aldershvile J. (1988). Independent effects of liver disease and chronic alcoholism on thyroid function and size: The possibility of a toxic effect of alcohol on the thyroid gland. Metabolism.

[50] Knudsen N., Bulow I., Laurberg P., Perrild H., Ovesen L., Jorgensen T. (2001). Alcohol consumption is associated with reduced prevalence of goitre and solitary thyroid nodules. Clin. Endocrinol..

[51] Livraghi T., Paracchi A., Ferrari C., Reschini E., Macchi R.M., Bonifacino A. (1994). Treatment of autonomous thyroid nodules with percutaneous ethanol injection: 4-year experience. Radiology.

[52] Rao N.L., Shetty S., Upadhyaya K., R.M P., Lobo E.C., Kedilaya H.P., Prasad G. (2010). Salivary C-reactive protein in Hashimoto’s thyroiditis and subacute thyroiditis. Int. J. Inflam..

[53] Baruah M.P., Bhattacharya B. (2012). Significant role of serum CRP in differentiating inflammatory from non-inflammatory causes of thyrotoxicosis. Indian J. Endocrinol. Metab..

[54] Pearce E.N., Bogazzi F., Martino E., Brogioni S., Pardini E., Pellegrini G., Parkes A.B., Lazarus J.H., Pinchera A., Braverman L.E. (2003). The prevalence of elevated serum C-reactive protein levels in inflammatory and noninflammatory thyroid disease. Thyroid.

[55] Boelen A., Maas M.A., Lowik C.W., Platvoet M.C., Wiersinga W.M. (1996). Induced illness in interleukin-6 (IL-6) knock-out mice: A causal role of IL-6 in the development of the low 3,5,3′-triiodothyronine syndrome. Endocrinology.

[56] Davies P.H., Black E.G., Sheppard M.C., Franklyn J.A. (1996). Relation between serum interleukin-6 and thyroid hormone concentrations in 270 hospital in-patients with non-thyroidal illness. Clin. Endocrinol..

[57] Moura Neto A., Zantut-Wittmann D.E. (2016). Abnormalities of thyroid hormone metabolism during systemic illness: The low T3 syndrome in different clinical settings. Int. J. Endocrinol..

[58] Ortiga-Carvalho T.M., Chiamolera M.I., Pazos-Moura C.C., Wondisford F.E. (2016). Hypothalamus-pituitary-thyroid axis. Compr. Physiol..

